# An Appended Domain Results in an Unusual Architecture for Malaria Parasite Tryptophanyl-tRNA Synthetase

**DOI:** 10.1371/journal.pone.0066224

**Published:** 2013-06-12

**Authors:** Sameena Khan, Ankur Garg, Arvind Sharma, Noelia Camacho, Daria Picchioni, Adélaïde Saint-Léger, Lluís Ribas de Pouplana, Manickam Yogavel, Amit Sharma

**Affiliations:** 1 Structural and Computational Biology Group, International Centre for Genetic Engineering and Biotechnology (ICGEB), New Delhi, India; 2 Institute for Research in Biomedicine (IRB Barcelona), Barcelona, Catalonia, Spain; 3 Catalan Institution for Research and Advanced Studies (ICREA), Barcelona, Catalonia, Spain; Griffith University, Australia

## Abstract

Specific activation of amino acids by aminoacyl-tRNA synthetases (aaRSs) is essential for maintaining fidelity during protein translation. Here, we present crystal structure of malaria parasite *Plasmodium falciparum* tryptophanyl-tRNA synthetase (Pf-WRS) catalytic domain (AAD) at 2.6 Å resolution in complex with L-tryptophan. Confocal microscopy-based localization data suggest cytoplasmic residency of this protein. Pf-WRS has an unusual N-terminal extension of AlaX-like domain (AXD) along with linker regions which together seem vital for enzymatic activity and tRNA binding. Pf-WRS is not proteolytically processed in the parasites and therefore AXD likely provides tRNA binding capability rather than editing activity. The N-terminal domain containing AXD and linker region is monomeric and would result in an unusual overall architecture for Pf-WRS where the dimeric catalytic domains have monomeric AXDs on either side. Our PDB-wide comparative analyses of 47 WRS crystal structures also provide new mechanistic insights into this enzyme family in context conserved KMSKS loop conformations.

## Introduction

Globally, malaria is still a serious health risk despite numerous concerted programs that aim to control it. Amongst the five species of *Plasmodia* that cause human malaria, *P. falciparum* causes most ill health and deaths [1]. Growing reports of malaria drug resistance therefore mandate constant new efforts at discovering novel protein targets for anti-malarial drug development. Over the past few years, several laboratories including ours have focused on components of the malaria parasite protein translation apparatus as new targets for inhibitors discovery [2]–[6]. The enzyme family of aminoacyl-tRNA synthetases (aaRSs) attaches specific amino acids to cognate tRNA molecules [7], [8]. Studies have revealed that aaRSs come in two flavors where each class shares sequence motifs and a similar topology in their catalytic domains [7], [8]. Class I aaRSs contain two conserved sequences - HIGH and KMSKS that trap ATP [7], [8]. Tryptophanyl-tRNA synthetase (WRS) is a class I aaRS and all members of this class are characterized by a Rossmann-fold catalytic domain whose active site is recognizable by the presence of consensus sequences HIGH and KMSKS [7]–[9]. The active site of Pf-WRS exhibits a third weakly conserved motif GIDQ (AIDQ in the human sequence) that is also involved in ATP binding. The key KMSKS loop has been found to be either ordered or disordered in individual crystal structures of WRSs from bacteria, human [10], [11].

Human WRS (Hs-WRS) has additional physiological roles in addition to aminoacylation [12]–[14]. Hs-WRS is secreted into the extracellular region of vascular endothelial cells and its splice variant form (mini-WRS) functions in vascular endothelial cell apoptosis as an angiostatic cytokine [15]. Hs-WRS is also the only aminoacyl-tRNA synthetase whose expression has been known to be induced by interferon (IFN-γ) [16], [17]. Amongst malaria parasite aaRSs, appendage of additional domains is common. Proteins which are homologous to the AlaRS editing domain named AlaX are found in many organisms [18], [19]. They are active in the *trans* hydrolysis of misacylated tRNA^Ala^ *in vitro* [18]. Crystal structures of AlaX-S (specific to Ser-tRNA^Ala^) and AlaX-M (specific to Ser-tRNA^Ala^ and Gly-tRNA^Ala^) from archaeon *Pyrococcus horikoshii* have been reported [20], [21]. Our earlier computational investigations had revealed that *P. falciparum* contains a variant WRS where the catalytic domain is fused to a distant homolog of the free-standing, proofreading factor AlaX [22], [23]. *Cryptosporidium parvum* genome also contains a single WRS gene that possesses an N-terminal AlaX like domain in addition to the conserved catalytic WRS domains [23].

The *Plasmodium falciparum* genome contains two copies of tryptophanyl-tRNA synthetase – a cytoplasmic version (hereafter Pf-WRS, Plasmodb ID PF13_0205) and an apicoplastic one (hereafter Pf-WRS^api^, Plasmodb ID PFL2485c) [3]. Here, we present crystal structure of malaria parasite *Plasmodium falciparum* cytoplasmic tryptophanyl-tRNA synthetase (Pf-WRS) catalytic domain (AAD) at 2.6 Å resolution in complex with L-tryptophan. Our confocal localization data show this enzyme to be present only in the parasite cytoplasm and exclude its secretion from the malaria parasite in asexual stages. Pf-WRS has an unusual N-terminal appendage of an Ala-X like domain (AXD) which we show is necessary for enzymatic activity. A construct of Pf-WRS that encodes for a protein segment that contains AXD and linker sequence of 72 residues is monomeric in solution. This suggests an unusual architecture for intact Pf-WRS. Finally, our PDB-wide comparative analyses of 47 WRSs give new mechanistic insights into this enzyme family in context of the conformation of the highly conserved KMSKS loops. Our comprehensive analyses therefore provide a platform for focusing on pathogen-specific WRSs for development of novel aaRS inhibitors.

## Materials and Methods

### Molecular cloning, expression and purification

Protein sequence for Pf-WRS was accessed from *Plasmodium* database (www.PlasmoDB.com) [24] and three gene constructs were made: 1) full length Pf-WRS encoding residues 1 to 631 (FLP), 2) N-terminal domain of residues 1–227 (NTD) and 3) catalytic domain of residues 228–631 which contains the aminoacylation and anticodon binding domains (AAD). All constructs were cloned into pETM-41 vector and transformed into *E. coli* B834 expression strain except for FLP which was transformed and expressed in *E. coli codon+* strain. Bacterial cell pellets for all three proteins was harvested after overnight growth at 18°C post-IPTG (isopropyl β-D-thioglactopyranoside, 0.5 mM) induction, and were suspended in a buffer containing 50 mM Tris (pH 7.5), 200 mM NaCl, 10 mM β-Me and protease inhibitor cocktail (Roche). All three proteins were purified using amylose resin and after buffer exchange (50 mM Tris (pH 7.5), 200 mM NaCl, 10 mM β-Me, 1 mM DTT & 0.5 mM EDTA) the MBP-tag was removed by overnight incubation (20°C) with TEV protease. The cleaved NTD & FLP were further purified using heparin chromatography and cation exchange chromatography (buffer exchange and binding in 25 mM MES (pH 6), 50 mM NaCl and elution in 25 mM MES, 1 M NaCl, pH 6), respectively. Gel filtration chromatography was performed for NTD and FLP on Superdex75 column (20 mM Tris, 100 mM NaCl, pH-7.1) and Superdex200 columns (20 mM Tris, 100 mM NaCl, pH-7.5) (GE) respectively. Best fractions were checked by SDS-PAGE and pure fractions were pooled and concentrated to using a 10 KDa centricon centrifugal device (Millipore) and stored at −80°C. The cleaved AAD was directly purified by GPC (20 mM Tris, 100 mM NaCl, pH-7.5) on Superdex200 column (GE healthcare). AAD protein was concentrated to 8.5 mg/ml using a 10 KDa centricon centrifugal device (Millipore) and stored at −80°C.

### Production of polyclonal antibodies and expression analysis

Polyclonal antibodies were raised in rabbits against highly purified recombinant Pf-WRS construct NTD and AAD protein. For western blot analysis, parasites in asynchronous *P. falciparum* cultures were released from infected RBCs (red blood cells) by 0.05%w/v saponin lysis and pellets were washed in phosphate buffered saline (PBS). The composition for 1× PBS was 8 g NaCl, 0.2 g KCl, 1.44 g Na_2_HPO_4_, 0.24 g KH_2_PO_4_ – pH 7.4. Parasites were lysed by 3 rounds of freeze-thawing in RIPA buffer (50 mM Tris-HCL, 150 mM NaCl, 1 mM EDTA, 1%v/v NP40 (nonyl phenoxypolyethoxylethanol), 0.1%w/v SDS, 1%w/v sodium deoxycholate, pH 7.4) containing protease inhibitors cocktail. Lysates were centrifuged and supernatants were separated on SDS-PAGE. Proteins were transferred to nitrocellulose membrane and blots were probed using specific primary antibodies and secondary alkaline phosphatase conjugated antibodies (1∶1500 dilutions). Bands were visualized using ECL detection kit, and parasite expressed Pf-WRS was probed using antibodies generated against recombinant Pf-WRS.

### Immunofluorescence assays using confocal microscopy

The IFA (Immunofluorescence assay) was performed as described earlier [25]. Cells were washed in PBS and fixed in solution using 4%w/v paraformaldehyde and 0.0075%w/v glutaraldehyde in PBS for 25 minutes. After a PBS wash, cells were permeabilized by using 0.1%v/v Triton X-100 in PBS for 10 minutes. After another PBS wash, cells were treated with 0.1 mg/ml sodium borohydride in PBS for 10 minutes. Cells were washed again with PBS, blocked in 3%w/v BSA in PBS for 1 hour and incubated overnight with protein-A column purified rabbit anti-protein IgG antibody (1∶250 dilution) at 4°C. Cells were then washed three times with PBS for 10 minutes each and incubated with Alexaflour 488–tagged anti-rabbit secondary antibody for 1 hour at room temperature and allowed to settle onto coverslips coated with poly-L-lysine (100 mg/ml). Finally, the coverslips were washed three times in PBS, mounted in antifade with DAPI reagent (Invitrogen) and sealed. Confocal microscopy was performed on a Nikon eclipse TE2000U microscope with a magnification of 100×. Pre-immune sera were taken as negative control.

### Aminoacylation assays and tRNA binding experiment

Aminoacylation of tRNA was performed at 37°C in 100 mM Hepes, pH 7.2, 20 µM tryptophan, 30 mM MgCl_2_, 30 mM KCl, 0.5 mM DTT, 5 mM ATP, 0.1 mg/ml BSA, 1000 Ci/mol L-[^3^H] tryptophan and 20 µM of Pf-tRNA^Trp^ transcripts. Reactions were initiated by addition of 2.5 µM pure Pf-WRS or Pf-WRS AAD enzyme and samples of 22 µl were spotted onto Whatman 3MM discs at varying time intervals. Radioactivity was measured by liquid scintillation. The Pf-tRNA^Trp^ (sequence from “Transfer RNA database”) was obtained by *in vitro* transcription using T7 RNA polymerase [26], [27]. For electrophoretic gel mobility-shift assay (EMSA), Pf-tRNA^Trp^ was labeled with γ-[^32^P] using T4 polynucleotide kinase and incubated for 20 min at 4°C with 15 mM MgCl_2_, 15 mM KCl, 0.5 mM DTT, 10% (w/v) glycerol, 75 mM Tris buffer pH 7.0, 20 ng/µl oligo(dT) and 5 µM of Pf-WRS, Pf-WRS AXD and Pf-WRS AAD. Competition assays were performed adding to the reaction 10-fold molar concentration of the non-radiolabeled *Plasmodium falciparum* transcribed tRNA^Trp^ or a heterologous tRNA (*E.coli* tRNA^Lys^ (Sigma)). Reactions were separated by electrophoresis onto a 6% (w/v) polyacrylamide gel in TBE 0.5×. Signal was digitalized using a PhosphoImagerTM from a gel exposed storage phosphor screen.

### Modeling, crystallization and structure determination

We generated atomic model of Pf-WRS AXD domain using *P. horikoshii* Ala-X like homolog structure (PDB: 1WNU) and the software MODELLER. Pf-WRS crystals were obtained at 20°C by hanging drop vapor diffusion method using 1 µl of Pf-WRS(8.5 mg/ml) and 1 µl of 0.09 M NPS (0.03 M NaNO_3_+0.03 M ammonium sulfate+0.0.3 M Na2HPO_4_), 0.1 M bicin pH 8.5, 12.5% MPD, 12.5%w/v PEG 1000, 12.5%w/v PEG 3350. Plate-shaped single crystals were directly mounted in cooled nitrogen gas at 100K. X-ray diffraction data were collected on a MARCCD detector at BM14 beam line of European Synchrotron Radiation Facility at Grenoble, France. The diffraction images were processed and scaled with *HKL2000* suite program [28]. Initial model was build by *AutoBuild* in PHENIX [29] which was subsequently rebuilt manually using COOT [30]. Model refinement was performed using *phenix.refine* in PHENIX. All the structural superimposition and preparation of figures was been carried out using UCSF chimera [31].

## Results

### Presence of atypical AlaX-like domain (AXD) in *P. falciparum* WRS

**Figure 1:**
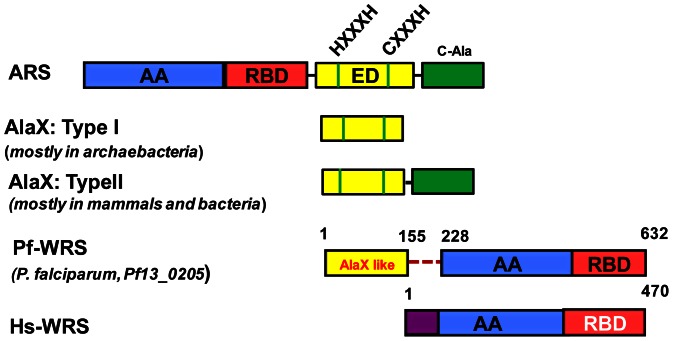
Domain structure and motifs in Pf-WRS, Hs-WRS, ARS and AlaX. The boundary regions of catalytic (blue), anticodon-binding (red), editing (yellow, green), and WHEP domains (purple) are shown. Conserved motifs of HXXXH and CXXH in ARS, AlaX (type I and II) are also shown. C-terminal extensions (green) in ARS and AlaX-II are marked. Note that the yellow domain (AlaX-like) is attached to catalytic domain of Pf-WRS with a 72 residue linker (red dashed line).

The N-terminal domain (NTD) of Pf-WRS is an extension of 227 residues which correspond to a domain spanning 1–155 (called AXD hereon) and a linker region from 156–227 (called linker hereon) (Figure 1). The Pf-WRS NTD (1–227 residues) is abundant in basic residues (∼12%), and bioinformatics analysis had suggested sequence similarity between its AXD and *Pyrococcus horikoshii* free standing type I AlaX domain [18], [23]. The AlaX-like appendage is not restricted to Pf-WRS but is present in other apicomplexans as well (*P. vivax*, *T. gondii*, *C. parvum* etc.). However, it is absent from the apicoplastic version of WRSs in apicomplexans. Despite reasonable sequence similarity between AXD and Type-I AlaXs, the conserved HXXXH and CXXXH motifs of AlaX family are not retained in AXD (Figure 1). Pf-WRS AXD is unlike Type II AlaX modules which have an additional C-terminal domain meant for specific contacts with tRNA^Ala^ [32]. Eukaryotic WRSs (like the human counterpart of Pf-WRS) contain specific N-terminal extensions called WHEP domains – these are helix turn helix structural motifs that play roles in RNA binding [33], [34]. Pf-WRS AXD however has no similarity to WHEP domains and AXD fusions to catalytic domains of WRSs are indeed unique chimeras.

### Expression and localization of *P. falciparum* WRS and its subdomains

**Figure 2:**
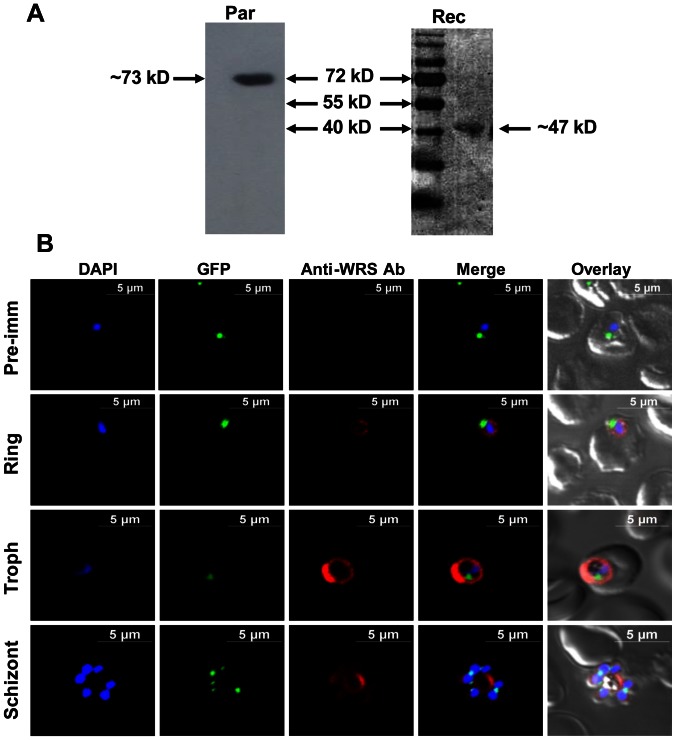
Expression and cellular localization of Pf-WRS AAD. (**A**). Pf-WRS (AAD) expression in parasites (Par) and detection of recombinant Pf-WRS (Rec) by western blot analysis. A larger size protein band was observed (∼73 kDa) in the parasite lysate consistent with gene annotation for (Pf-WRS, residues 1–632). The recombinant Pf-WRS AAD migrated at ∼46.8 kDa as expected from the truncated DNA construct (Pf-WRS AAD, residues 228–632) (**B**). Localization of Pf-WRS in asexual parasite stages. Parasite line was GFP-tagged (strain D10 ACP_leader_-GFP) where apicoplast fluorescence is in green. DAPI staining is in blue while Pf-WRS is stained with Alexa594 (red). Pre-immune sera was used as negative control in these experiments.

We ruled out the possibility of an alternative translation start site in gene for Pf-WRS using antibodies specific to Pf-WRS subdomains and by interrogating the size of natively expressed Pf-WRS in malaria parasites. Pf-WRS (AAD) antibodies were tested against parasite lysates and the quality of recombinant Pf-WRS was confirmed by western blot analysis where both lysate and recombinant material revealed one band for Pf-WRS but at expectedly different sizes (Figure 2A). A larger size band was observed (∼73 kD) in the parasite lysate in line with gene annotation for (Pf-WRS, residues 1–632). The recombinant Pf-WRS AAD migrated at ∼46.8 kD as expected from the truncated DNA construct (Pf-WRS AAD, residues 228–632) (Figure 2A). No smaller Pf-WRS protein corresponding either to the catalytic domain (AAD) or AXD was observed. Further, the parasites were co-stained with DAPI and GFP marker (for apicoplast) to exclude presence of Pf-WRS from parasite nucleus and its apicoplast (Figure 2B). These data clearly show cytoplasmic localization of Pf-WRS in all the asexual life stages of the parasite (Figure 2B) suggesting that this enzyme contributes to production of tryptophanyl-adenylate in parasite cytoplasm (and is distinct from the apicoplastic WRS activity). The cytoplasm localized Pf-WRS showed evenly spread staining in the parasites as judged by visual analyses and volumetric examination of z-stacks by confocal microscopy. Confocal IFAs with pre-immune sera and anti-histidine tag antibodies failed to produce fluorescence, validating the specificity of Pf-WRS antibodies (Figure 2B). No signals were seen for Pf-WRS at the infected RBC membrane under normal growth conditions suggesting lack of Pf-WRS secretion from the parasite. These localization studies suggest differences between Pf-WRS and Hs-WRS in terms of non-canonical localizations of WRSs.

### Molecular modeling of AXD and the overall architecture of Pf-WRS

**Figure 3:**
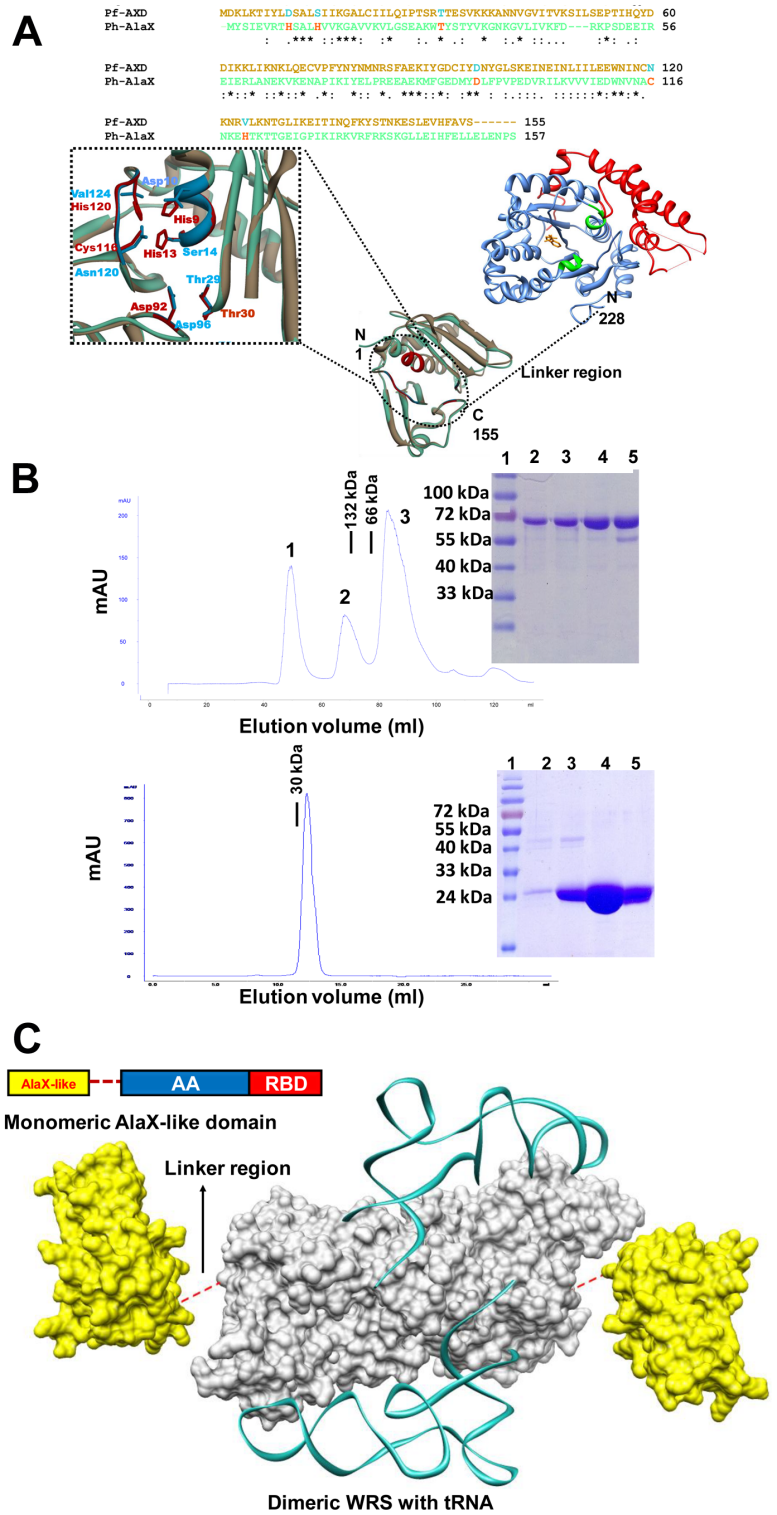
Domain architecture, structural modeling and gel permeation chromatography of Pf-WRS. (**A**). Sequence alignment of AlaX-like domain present in N-terminal region of Pf-WRS with AlaX (top). The critical active site residues of AlaX are shown but are not conserved in AXD. Three-dimensional model for Pf-WRS AXD is shown attached via a linker to main protein body (**B**). Size exclusion chromatography profile for full length Pf-WRS (residue 1–632, dimeric molecular weight ∼140 kDa) is shown on top panel. Protein purification was monitored by using UV (280 nm) absorbance in all experiments. The three major protein peaks in correspond to 1) insoluble protein aggregates, 2) Pf-WRS (which migrates as a dimer) and 3) cleaved MBP-fusion tag (migrates as a monomer of 42K) respectively. The bottom panel displays migration of NTD at expected molecular weight and as a monomer. In both cases, SDS-PAGE shows full length Pf-WRS (4 lanes, single bands of ∼73 kDa) in upper panel, and NTD (single band of ∼26 kDa) in lower panel. These fractions were recovered as peaks in the gel filtration runs. The molecular weight markers are BSA (132 kDa and 66 kDa) and DTD (30K) shown by black bars. Gel filtration chromatography was performed for NTD on Superdex75 column (void volume - 7 ml) and for FLP on Superdex200 column (void volume - 40 ml) respectively (**C**). Modeling of the overall architecture of full length Pf-WRS where monomeric NTDs (yellow) are attached to both to the dimeric catalytic cores (grey). Wire diagram shows modeled tRNA based on crystal structure of human-WRS in complex with tRNA.

In the absence of an atomic structure for AXD (or NTD), we built a 3D model by homology modeling approaches using AlaX structure from *P. horikoshii* (PDB: 1WNU). It is evident that AXD is not directly fused to WRS main enzyme body and instead is tethered via a 72 residue linker (Figure 3A). Modeling and secondary structure predictions of Pf-WRS AXD suggested α+β structure as seen in orthologous bacterial and eukaryotic AlaX enzymes (Figure 3A). A closer inspection of 3D model of AXD superimposed on *Ph* AlaX-S revealed that the zinc binding residues in latter (His-9, His-13, Cys-116 and His-120) are replaced by Asp-9, Ser-13, Asn-116, and Val-120 in AXD (Figure 3A). Therefore, Pf-AXD is unlikely to retain the hydrolytic activity associated with AlaX-S and may not function as an editing domain in malaria parasites.

We recombinantly produced and purified proteins corresponding to Pf-WRS, NTD and AAD subdomains. These proteins were tested for their oligomeric status using size exclusion chromatography. Interestingly, the NTD module migrated as a monomeric species while Pf-WRS and AAD were dimeric in solution (Figure 3B). Migration of the full length Pf-WRS is shown in the figure 3B (upper panel). The three major peaks in gel filtration chromatogram of full-length Pf-WRS correspond to insoluble protein aggregates (just after the void volume of the column i.e. 40 ml), full length WRS (which migrates as a dimer) and cleaved MBP-fusion tag (migrates as a monomer of ∼42K). SDS-PAGE analyses show full length Pf-WRS in all four lanes (single bands of ∼73 kDa) in the upper panel. The domain NTD (single band of ∼26 kDa) is evident in the lower panel (Figure 3B). These analyses suggested that Pf-WRS likely has a unique overall architecture where the monomeric AXDs are linked to main protein body via the 72 residue linker sequence that runs into the dimeric Pf-WRS AAD (Figure 3C). We further modeled binding of tRNA to WRS using the homologous human WRS-tRNA crystal structure (PDB: 2AKE). This revealed a possible arrangement of Pf-WRS subdomains in context of the overall structure of Pf-WRS (Figure 3C). We next tested the significance of NTD in terms of tRNA binding and aminoacylation activity of Pf-WRS.

### tRNA binding and aminoacylation activity assays

**Figure 4:**
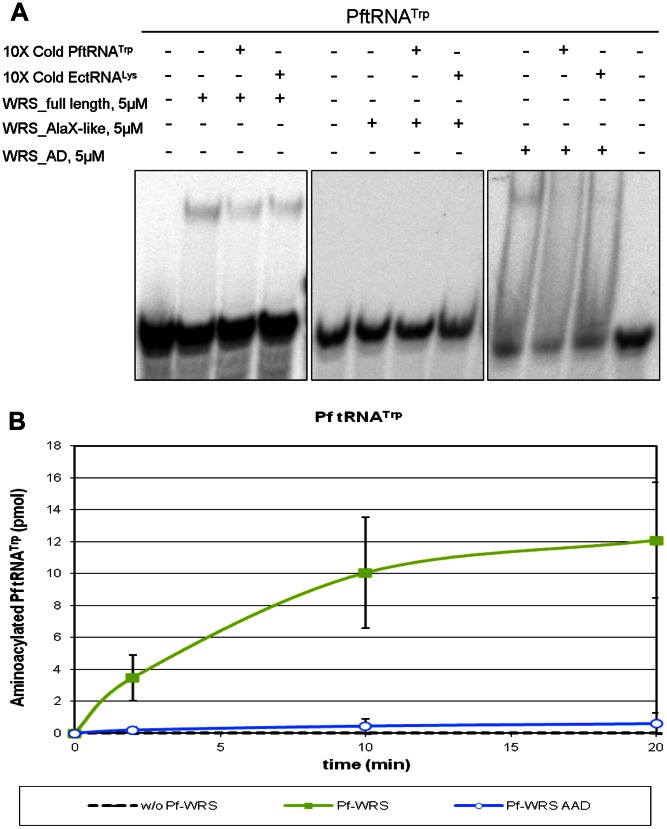
tRNA binding and aminoacylation assays with Pf-WRS and its subdomains. (**A**). EMSA competition experiments performed with labeled Pf-tRNA^Trp^ and 5 µM of Pf-WRS, Pf-WRS AXD or Pf-WRS AAD (referred to as AD in the figure). Competitors are unlabeled Pf-tRNA^Trp^ or heterologous tRNA (*E. coli* tRNA^Lys^) and were added at a 10-fold excess. The addition of cold Pf-tRNA^Trp^ does indeed influence the amount of observed shift in the gel retardation assays (compare intensity of the shifted (upper) band in lane 2 to those of in lanes 3 and 4 where the same size shifted band is clearly less intense (implying that addition of 10× cold Pf-tRNA^Trp^ has indeed reduced the amount of complex formed between full length Pf-WRS and tRNA. No evident competition with cold Pf-tRNA^Trp^ in experiments with Pf-WRS catalytic domain (lanes 10 and 11 in comparison with lane 9) suggests that Pf-WRS catalytic domain does not appreciably or significantly bind tRNA in a specific manner (**B**). Aminoacylation assay of Pf-WRS (green line) and Pf-WRS AAD (truncated form without AXD, blue line) performed with Pf-tRNA^Trp^ at 37°C. A control without enzyme (dash line) was added. The amount of enzyme per assay was 2.5 µM and 5 µM of Pf-tRNA^Trp^. Each time points were counted by scintillation.

We tested whether the aminoacylation activity of Pf-WRS required the participation of the two domains of the protein (AXD and AAD) or, as in other cases, just the conserved aminoacylation domain (AAD) was sufficient. As can be seen in figure 4B, the purified full length enzyme was capable of efficiently charging Pf-tRNA^Trp^, but the enzyme lacking AXD does not display aminoacylation activity. This result suggests that AXD potentially contributes to the binding of Pf-tRNA^Trp^, or that it was structurally important for the catalytic activity of the enzyme. To address this question, we performed electrophoretic mobility shift assays (EMSA) to determine if Pf-WRS AXD was capable of binding Pf-tRNA^Trp^. As can be seen in figure 4A, AXD is not capable of retarding the mobility of Pf-tRNA^Trp^. By contrast, the full-length enzyme and the AAD both reduced the mobility of their cognate tRNA substrates. These results suggest that the essential role in aminoacylation of AXD likely stems from structural contributions to the active site domain of Pf-WRS.

### Crystal structure of Pf-WRS catalytic domain (AAD)

**Figure 5:**
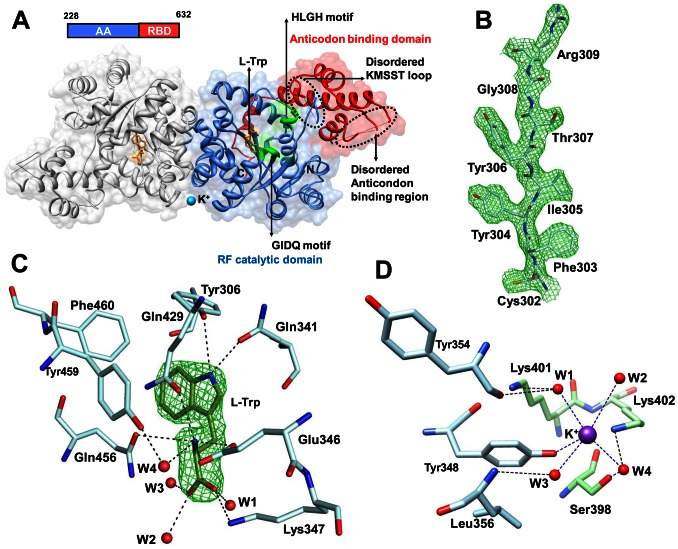
Crystal structure analysis of Pf-WRS AAD in complex with L-Trp. (**A**). Bound tryptophan (L-Trp, orange) and K^+^ ion (blue sphere) are also shown. The aminoacylation domain (blue), anticodon binding domains (red) and the conserved motifs HLGH and GIDQ (green) are highlighted for one monomer. The other monomer is shown in grey. Disordered KMSST loop and anticodon binding region are indicated by dashed circles (**B**). A portion of the model showing the quality of the 2Fo-Fc electron density map which is contoured at 2.0 σ level (**C**). Difference Fourier map (Fo-Fc) showing the bound substrate (L-Trp, green). The map is contoured at 5σ level. Interactions between L-Trp, protein residues (blue) and water molecules (red sphere) are indicated by dashed lines (**D**). Bound potassium ion, coordinating residues from each monomer and water molecules are shown.

**Figure 6:**
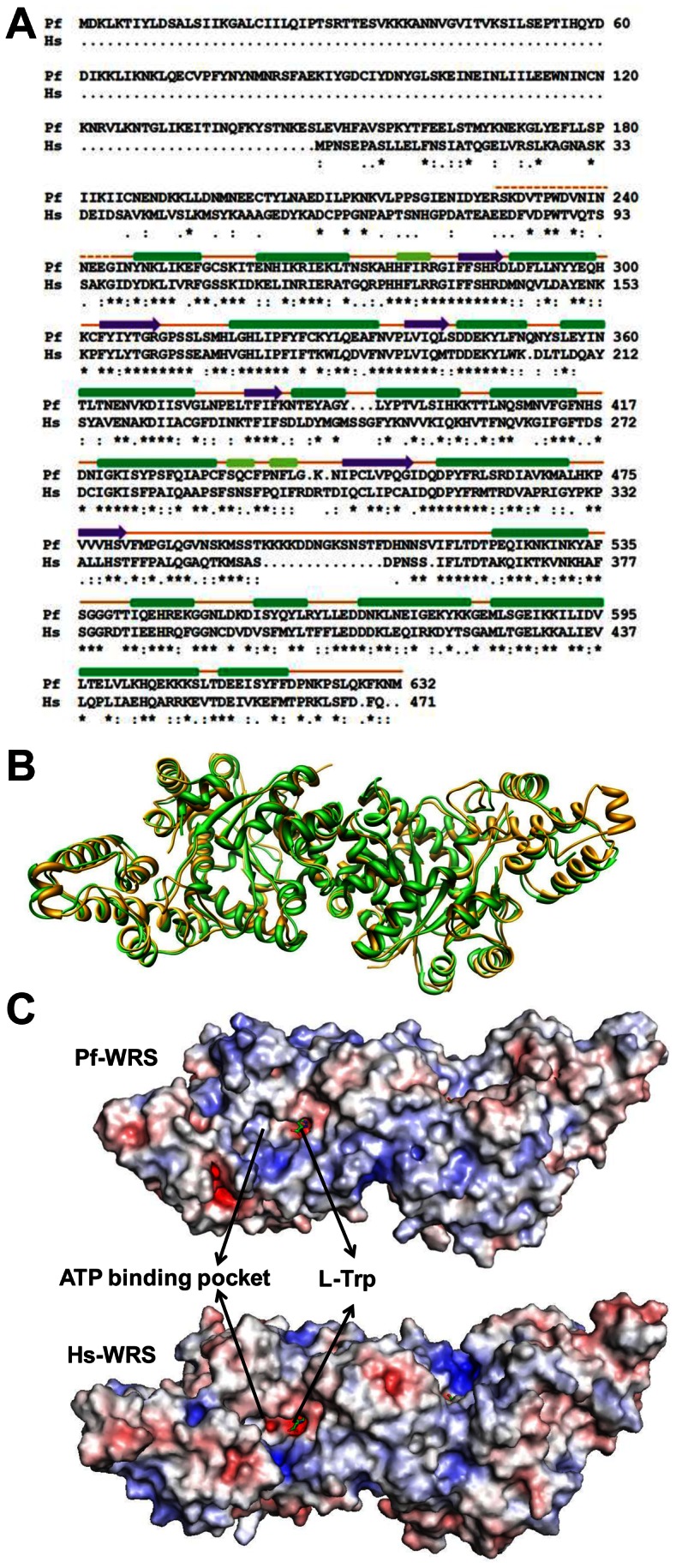
Sequence, structure, electrostatic and motif comparisons between Pf-WRS and Hs-WRS. (**A**). Highly conserved residues, conserved ones, and semi-conserved residues are marked with asterisks, semicolons and dots respectively. The three conserved motifs HLGH, GIDQ and KMSST are shown in red boxes. L-Trp (red), K^+^ ion (blue) interacting residues and insertion residues (purple) in KMSST loop are highlighted (**B**). Superposition of Pf-WRS dimers (green) with Hs-WRS (gold) shows the high degree of structural conservation in the catalytic domains of each (**C**). Comparison of electrostatic surfaces of Pf-WRS and Hs-WRS. The electrostatic surfaces are displayed as color gradients in red (electronegative, ≤−10 kTe^−1^) and blue (electropositive, ≥10 kTe^−1^) (**D**). Left panel shows superposition of two human-WRS structures in red (105T) and green (1ULH). In both cases, the ATP binding pocket is devoid of ligand and the KMSAS loop (in blue) is ordered. Right panel shows superposition of two human-WRS structures in red (105T) and green (2QUI) where the former is in apo form whereas the latter has ATP (in yellow) bound. Once again, the KMSAS loop (in blue) is ordered in both cases.

We were unable to get crystals for full length Pf-WRS (1-632) and NTD (1-227). However, a construct encoding for the catalytic domain (AAD) of Pf-WRS (228–632, Figure 5A) crystallized in the presence of L-tryptophan (L-Trp). These crystals diffracted to 2.6 Å resolution and belong to space group P2_1_ with two dimers of Pf-WRS AAD in the asymmetric unit (solvent content of ∼56%). Pf-WRS AAD structure was solved by molecular replacement using crystal structure of human WRS (PDB: 2QUH) as the search model (Figure 5A, Table 1). The final electron density is well defined in all four chains except for N-terminal residues 228–244 and for KMSST loop residues 486–512 (Figure 5B). Structure of Pf-WRS AAD (monomer) consists of 6 β strands and 19 α helices and each monomer harbors the N-terminal aminoacylation domain (residues 228–496) and an anticodon-binding domain (residues 497–629, Figure 5A). Well defined electron density was observed for the bound L-Trp in the active site of all four chains (Figure 5C). A potassium ion (K^+^ ion) was observed at the dimer interface (Figure 5D) and the coordination distances between this ion, protein atoms and water molecules are in ranges of 2.8–3.6 Å. Residues from both monomers contribute to the ligation of the bound K^+^ ion, which to our knowledge has not been reported for any WRS crystal structure so far. The Rossmann fold catalytic domain of Pf-WRS contains the conserved HLGH and GIDQ motifs (Figure 5A, 5C). Sequence identity between parasite and human enzyme counterparts is ∼55% and the overall fold of Pf-WRS is hence very similar to Hs-WRS (Figure 6A, 6B). However, the KMSST loop in Pf-WRS has a fifteen residues insertion immediately following this key motif (Figure 6A). The significance of this insertion at this location is unclear, and sequence analyses of WRSs suggest that the occurrence of this insertion is unique to malarial WRSs. The human and Pf-WRS AAD dimers superimpose with RMSD of ∼1.5 Å for 689 C^α^ atoms (Figure 6B). No significant difference in the overall electrostatic surface features between Pf-WRS and Hs-WRS was evident (Figure 6C).

**Table 1:** Data collection and refinement statistics.

*Data collection*	
Wavelength (Å)	0.97625
Crystal-to-detector distance (mm)	270
Exposure time (seconds)	60
Number of frames	360
Unit-Cell parameters (Å, °)	a = 51.176, b = 213.252 , c = 101.952; α = 90, β = 97.15, γ = 90
Space group	P2_1_
Resolution (Å)	50.0-2.60 (2.64-2.60)
Unique reflections	62582 (3006)
Multiplicity	6.9 (6.7)
Completeness (%)	94.4 (91.3)
<I/σ(I)>	24.9 (2.9)
R_merge_	0.075 (0.585)
*Refinement*	
Resolution (Å)	47.8-2.60
Reflections in work set/test set	61254/1274
R_work_/R_free_ (%)	19.2/22.9
*Model composition*	
No. of protein residue/waters	1435/239
L-Trp/BME/K^+^ ion	4/3/2
*Stereochemistry*	
RMSD in bond lengths (Å)	0.004
RMSD in bond angles (°)	0.855
*Ramachandran plot, residues in (%)*	
Most favoured regions	90.5
Additionally allowed regions	9.5
*Mean B factors (Å^2^)*	
Protein atoms/waters	55.0/38.9
L-Trp/BME/K^+^ ion	32.8/68.5/62.5

Values in parentheses are for the highest resolution shell.

### Conformation of ATP binding KMSKS loops in WRSs

In Pf-WRS AAD crystal structure residues 482–512 include the ATP-binding motif KMSST which is completely disordered. We could only observe clear electron density for the bound L-Trp but not for ATP although crystals were grown in the presence of both. Bacterial WRS is known to bind ATP that involves reordering of its KMSKS loop region [10]. On the other hand, Hs-WRS exhibits a closed conformation of the active site in which the KMSAS loop can be ordered even in the absence of ATP [11]. We therefore decided to study the conformation of all KMSKS loops in the 47 WRS crystal structures available in PDB with a view to cataloging KMSKS conformations as a function of ligand binding in the ATP binding pocket of WRSs (Table 2). This analysis suggested that in ∼81% of the apo WRS structures the KMSKS loop is highly ordered despite absence of ATP (Table 2 and Figure 6D). ATP occupancy in the active site therefore does not necessarily correlate with a ‘closed’ conformation for KMSKS motif in WRSs (Figure 6D).

**Table 2:** List of WRS structures available in PDB.

No.	PDB	Organism	W/analog pocket	ATP/mimic pocket	W-interacting residues	KMSKS loop	Other ligands
1.	4JFA	*P. falciparum*	Holo	Apo	-	Disordered	Potassium
2.	1O5T	*H.sapiens*	Apo	Apo	Identical	Ordered	-
3.	2AKE	*H.sapiens*	Holo	Apo	Identical	Ordered	Sulfate, tRNA
4.	2DR2	*H.sapiens*	Holo	Apo	Identical	Ordered	Sulfate, tRNA
5.	2QUH	*H.sapiens*	Holo	Apo	Identical	Ordered	-
6.	2QUI	*H.sapiens*	Holo	Holo-ATP	Identical	Ordered	Magnesium, LTN
7.	2QUK	*H.sapiens*	Apo	Apo	Identical	Ordered	-
8.	2QUJ	*H.sapiens*	Holo	Holo-AMP	Identical	Ordered	-
9.	1ULH	*H.sapiens*	Apo	Apo	Identical	Disordered	-
10.	1R6U	*H.sapiens*	Holo	Holo	Identical	Ordered	-
11.	2AZX	*H.sapiens*	Holo	Apo	Identical	Ordered	Magnesium, Sulfate
12.	1R6T	*H.sapiens*	Holo	Holo	Identical	Ordered	-
13.	3A05	*A.pernix*	Holo	Apo	Non-identical	Ordered	-
14.	3A04	*A.pernix*	Apo	Apo	Non-identical	Ordered	Fe-S cluster, Cadmium
15.	2OV4	*B.stearothermophilus*	Apo	Ap4A	Non-identical	Ordered	-
16.	3PRH	*B.subtilis*	Apo	Apo	Non-identical	Ordered	-
17.	3M5W	*C.jejuni*	Apo	Apo	Non-identical	Ordered	Sulfate
18.	3TZL	*C.jejuni*	Holo	Holo-ADP	Non-identical	Ordered	-
19.	3HV0	*C.parvum*	Holo	Apo	Identical	Disordered	-
20.	1YID	*D.radiodurans*	Apo	Holo	Non-identical	Ordered	Magnesium
21.	1YIA	*D.radiodurans*	Holo (5-OH)	Apo	Non-identical	Ordered	-
22.	1YI8	*D.radiodurans*	Holo	Apo	Identical	Ordered	-
23.	2A4M	*D.radiodurans*	Holo	Apo	Non-identical	Ordered	-
24.	3TZE	*E.cuniculi*	Holo	Apo	Identical	Ordered	-
25.	3HZR	*E.histolytica*	Apo	Apo	Non-identical	Ordered	-
26.	3FOC	*G.lamblia*	Apo	Apo	Identical	Ordered	Sulfate
27.	1I6M	*G.stearothermophilus*	Apo	Apo	Non-identical	Ordered	Sulfate
28.	1I6L	*G.stearothermophilus*	Apo	Apo	Non-identical	Ordered	Sulfate, Ammonium
29.	1I6K	*G.stearothermophilus*	Holo	Holo-AMP	Non-identical	Ordered	Sulfate, Ammonium
30.	1D2R	*G.stearothermophilus*	Apo	Apo	Non-identical	Ordered	-
31.	1M83	*G.stearothermophilus*	Apo	Holo	Non-identical	Ordered	Magnesium
32.	1MAU	*G.stearothermophilus*	Holo	Holo	Non-identical	Ordered	Magnesium, LTN
33.	1MB2	*G.stearothermophilus*	Holo	Apo	Non-identical	Ordered	-
34.	1MAW	*G.stearothermophilus*	Apo	Holo	Non-identical	Ordered	-
35.	3FI0	*G.stearothermophilus*	Holo	Holo-AMP	Non-identical	Ordered	Phosphate
36.	3FHJ	*G.stearothermophilus*	Holo	Holo-AMP	Non-identical	Ordered	Phosphate
37.	2YY5	*M.pneumonia*	Holo	Holo	Non-identical	Ordered	Sulfate
38.	3JXE	*P.horikoshii*	Holo	Holo-AMP	Identical	Ordered	Sulfate
39.	3KT0	*S.cerevisiae*	Apo	Apo	Identical	Disordered	EMN
40.	3KT3	*S.cerevisiae*	Holo	Holo-AMP	Identical	Ordered	Sulfate
41.	3KT6	*S.cerevisiae*	Holo	Apo	Identical	Ordered	Sulfate
42.	3KT8	*S.cerevisiae*	Holo	Apo	Identical	Ordered	Sulfate, LTN
43.	2IP1	*S.cerevisiae*	Apo	Apo	Identical	Disordered	-
44.	3I05	*T.brucei*	Apo	Apo	Identical	Disordered	-
45.	2G36	*T.maritime*	Holo	Apo	Non-identical	Ordered	Fe-S cluster
46.	2EL7	*T.thermophilus*	Apo	Apo	Non-identical	Ordered	Phosphate
47.	3SZ3	*V.cholera*	Holo	Apo	Non-identical	Ordered	-
48.	3N9I	*Y.pestis*	Apo	Apo	Non-identical	Ordered	Calcium

The apo/holo forms and KMSKS loop order are shown.

## Discussion

*P. falciparum* encodes 36 aminoacyl-tRNA synthetases (aaRSs) of which 15 are apicoplastic and 17 cytoplasmic [3], [22]. Another 4 aaRS are dual localized to parasite cytoplasm and apicoplast [3], [22]. Despite these recent advances in our understanding of malarial aaRSs, structural information on only two malarial aaRSs is so far available [2], [4]. Several groups are presently actively engaged in exploring the potential of inhibiting parasite aaRSs as new targets for antimalarial drug development [2]–[4], [6]. In this vein, we present crystal structure of cytoplasmic tryptophanyl-tRNA synthetase at 2.6 Å resolution. Our comprehensive structure, functional and bioinformatics analyses provide new insights into pathogen-specific WRSs.

Using confocal microscopy data, we confirm cytoplasmic localization of Pf-WRS and exclude the possibility of its secretion from the parasite into infected human red blood cells. Pf-WRS has an unusual architecture as it is a fusion of WRS catalytic domain to a module termed here as AXD which resembles AlaX editing domains [18], [35]. Latter are editing domains which hydrolyze tRNA^Ala^ misacylated with Ser/Gly [18], [35]. Based on analysis of genomic databases, we observed that except for its presence in brown alga *Ectocarpus siliculosus*, AXD-fused tryptophanyl-tRNA synthetase is only found in pathogen lineages. We identified AXD-WRS fusion in the following alveolates: *Plasmodia* (mammals), *Toxoplasma gondii* (mammals), *Cryptosporidium parvum* (mammals), *Babesia bovis* (mammals), *Neospora caninum* (mammals), *Theileria annulata* (mammals) and the *dinoflagellate Perkinsus marinus* (oyster). However, in case of *P. falciparum* AXD the crucial residues required for hydrolytic activity are mutated, and therefore this domain in unlikely to retain its editing activity. Intriguingly, malaria parasite genomes do not encode for a free standing AlaX homolog, and therefore it remains unclear how these parasites ensure fidelity after mischarging of tRNA^Ala^ [22].

We also show that NTD region of Pf-WRS is not only required for binding to tRNA^Trp^ but also for aminoacylation activity of Pf-WRS (Figure 4A). These results are in contrast to studies with *C. parvum* WRS which have earlier shown that its N-terminal extension is dispensable for enzymatic activity [23]. Our analyses of oligomeric structures of Pf-WRS subdomains indicate that the overall architecture of Pf-WRS is likely to be atypical (Figure 3B). The dimeric catalytic domains of Pf-WRS likely each have an attachment of AXDs with 72 residue linkers on each end (Figure 3C). There are no reports of mischarging of tRNA^Trp^ by WRSs, and therefore presence of AlaX-like domain fusions with catalytic domains of WRSs may simply provide additional tRNA binding capabilities to these enzymes. Our tRNA binding and aminoacylation data are therefore consistent with the idea that these WRS N-terminal extensions assist in aminoacylation activity rather than in proofreading.

Comparative structural analysis of Pf-WRS highlights unique features in the *Plasmodium* enzyme when compared to its human counterpart. Pf-WRS contains a 15 residue insertion adjacent to the highly conserved KMSKS (KMSST in Pf-WRS) loop. Given the conformational plasticity of KMSKS loops in class I tRNA synthetases, the significance of a lysine-enriched 15 residue insertion in Pf-WRS remains unclear. Our structural analyses of 119 WRS monomeric chains from 47 deposited WRS crystal structures from archaeal/bacterial/parasitic/human sources reveal intriguing details of the inherent flexibility of KMSKS loop irrespective of ATP binding (Table 2 and Figure 6D). We analyzed 47 WRS structures from the PDB server. Of these, 32 were apo WRSs (see Table 2) where we analyzed at least one monomer each. In these, the KMSKS loop is disordered in 6 (∼19%) while it is ordered in 26 (∼81%). Clearly, in majority (81%) of the cases therefore ATP occupancy in the active site is not a prerequisite for the KMSKS loop to adopt a distinct, ordered structural state – the so called ‘closed’ conformation (Table 2 and Figure 6D). We also found no evidence of large conformational changes between any of the L-Trp analogs, substrate analogs and AMP/ADP/ATP or ATP+L-Trp analogs in the bound/unbound states of ∼119 WRS chains available in the PDB. Finally, we observed a unique potassium binding site at the Pf-WRS AAD dimer interface, and note that the ligating residues are not conserved between malarial and human enzymes. In summary, our analyses highlight Pf-WRS as essentially an alveolate-specific gene fusion of WRS catalytic and AlaX-like domains where the latter assists in tRNA binding and aminoacylation reactions. These new insights may potentially be exploited for structure-guided development of novel aaRS inhibitors.

### Accession Number

Coordinates and structure factors for Pf-WRS AAD have been deposited in the RCSB Protein Data Bank under accession code 4JFA.
